# Clinical value of mean platelet volume in predicting and diagnosing pre-eclampsia: a systematic review and meta-analysis

**DOI:** 10.3389/fcvm.2023.1251304

**Published:** 2023-10-06

**Authors:** Dan Ye, Shuwen Li, Yi Ding, Zhenqin Ma, Rongxia He

**Affiliations:** ^1^ The Second Clinical Medical College, Lanzhou University, Lanzhou, China; ^2^ Department of Obstetrics, Lanzhou University Second Hospital, Lanzhou, China

**Keywords:** mean platelet volume, prevention, diagnosis, pre-eclampsia, meta-analysis

## Abstract

**Background:**

Pre-eclampsia (PE) is a severe pregnancy complication. Thrombocytopenia and platelet dysfunction are common hematology disorders in PE. Previous studies considered mean platelet volume (MPV), a functional marker of platelets, as a potentially useful predictor for the diagnosis of PE.

**Methods:**

PubMed, China Biomedical Literature Database, Chinese National Knowledge Infrastructure, Embase, Wanfang, VIP, and Cochrane Library databases were searched to gather diagnostic trials evaluating the diagnosis of PE using MPV, from their inception to 13 March 2023. We also searched Google Scholar and Baidu.

**Results:**

A total of 22 studies from 20 articles were found. The pooled diagnostic accuracy of the MPV for PE recognition was as follows: sensitivity (SEN) 0.676 [95% confidence interval (CI) (0.658–0.694)], specificity (SPE) 0.710 [95% CI (0.703–0.717)], and diagnostic odds ratio (DOR) 7.012 [95% CI (4.226–11.636)], and the SROC-AUC and Q* indices were 0.7889 and 0.7262, respectively. The pooled SEN, SPE, and DOR of the diagnostic accuracy of MPV for PE before 16 weeks of gestation were 0.707 [95% CI (0.670–0.743)], 0.639 [95% CI (0.611–0.667)], and 4.026 [95% CI (2.727–5.943)], and the SROC-AUC and Q* indices were 0.7278 and 0.6753, respectively. For the interval of truncation values between 9 and 10 fl, the SROC-AUC and Q* indices for MPV were 0.8856 and 0.8162, respectively.

**Conclusions:**

Available evidence suggests that MPV has a moderate predictive and diagnostic value for PE, particularly in diagnosing after 20 weeks of gestation. The diagnostic accuracy is higher when the MPV cut-off falls between 9 and 10 fl. The sensitivity of MPV alone in diagnosing PE is not high, and the combination of other markers for predictive diagnosis may better differentiate PE.

**Systematic Review Registration:**

https://www.crd.york.ac.uk/prospero/display_record.php?ID=CRD42023425154, identifier: CRD42023425154.

## 1. Introduction

Maternal mortality is a serious global problem. In 2019, WHO (World Health Organization) reported that almost 95% of maternal deaths occur in low- and lower–middle-income countries, in which pre-eclampsia (PE) and eclampsia are important causes (1). Each year, more than 500,000 fetal and neonatal deaths and more than 70,000 maternal deaths occur worldwide because of PE (2). PE manifests primarily as a progressive pregnancy disorder with new-onset hypertension occurring after 20 weeks of gestation with the simultaneous involvement of multiple organ systems (3, 4). Clinical studies have confirmed that PE increases the risk of developing chronic diseases in later life, including cardiovascular complications (5, 6), renal disease (7), and neurological disorders (8). At the same time, PE is associated with neonatal neurodevelopmental problems (9) and congenital heart disease (10).

The etiology of PE is unclear, and its pathophysiological mechanisms are associated with placental hypoperfusion, endothelial dysfunction, oxidative stress, inflammation, and immune abnormalities (4). Pregnancy changes the maternal hemostatic–fibrinolytic system, shifting the equilibrium toward a hypercoagulable state. However, PE exacerbates this change process (11, 12). Endothelial cell dysfunction leading to vasoconstriction and platelet adhesion aggregation, triggering coagulation, increased platelet activation leading to increased platelet consumption, and subsequent stimulation of the inflammatory response are key pathogenic steps in PE, causing thrombocytopenia, a prevalent hematological abnormality in PE (13–15).

Mean platelet volume (MPV), a platelet-related index, is a marker of platelet size, function, and activation. During platelet activation, the number and size of pseudopods will increase, while platelet depletion leads to the release of new and larger platelets, resulting in an increment in MPV (16, 17). MPV is a non-invasive biomarker. Compared with other plasma or serum-based biomarkers and various imaging modalities for prediction, MPV allows the use of complete blood count (CBC) tests in limited healthcare resources, which is simpler and less costly, reducing the healthcare burden on pregnant women in low- and middle-income areas. It is demonstrated that MPV can be used as a predictor of the severity and prognosis of cardiovascular disease (18, 19) and infectious diseases (20), while increased MPV is associated with the occurrence and severity of gestational diabetes (21) and intrahepatic cholestasis (22).

Drugs that significantly slow the progression of PE have not been identified, and the only option to prevent the disease is to deliver the fetus and placenta. Aspirin is the only preventive medication for PE strongly supported by research evidence (23). National guidelines recommend that women with high-risk factors can start taking aspirin before 16 weeks of gestation (24–26).

Aspirin operates by regulating vascular homeostasis and platelet function (26). Therefore, understanding the variation in MPV and its associated predictive value in PE is increasingly crucial. Predicting PE in early gestation, identifying women most suitable for aspirin prophylaxis, and eliminating the short- or long-term adverse consequences caused by PE remain a challenge, especially when considering the conditions of scarce medical resources in low- and middle-income areas. Evidence from cohort studies and meta-analyses indicates that PE is associated with elevated platelet function, and MPV, an important parameter of platelet activation, is elevated in PE (14–16, 27, 28). Further evaluation is needed to determine whether MPV can be used as a marker for the early diagnosis of PE. This meta-analysis aims to initially accumulate the available literature to assess the diagnostic efficacy of MPV as a predictive and diagnostic marker for PE.

## 2. Methods

This meta-analysis was based on the Preferred Reporting Items for Systematic Reviews and Meta-Analyses (PRISMA) guidelines (29), and registration was completed with PROSPERO (CRD42023425154). We included diagnostic trials to investigate the predictive and diagnostic value of MPV for PE in pregnant women across all trimesters.

### 2.1. Search strategy

Two authors (DY and SL) independently performed the database search. The PubMed, Cochrane Library, Embase, Chinese National Knowledge Infrastructure (CNKI), Wanfang, China Biomedical Literature Database (CBM), and VIP databases were searched to gather publicly published articles from the establishment of the database to 13 March 2023. In addition, Google Scholar, Baidu, and the references of the included literature were also searched to supplement access to relevant literature. Ages, races, gestational weeks, and languages were not limited in the selection process. A medical subject headings (MeSH) thesaurus in conjunction with free word, in combination with Boolean operators (e.g., “OR” or “AND”), was used. The search terms included Mean Platelet Volume, Mean Platelet Volumes, Platelet Volume, Mean, Volume, Mean Platelet, MPV, blood indices, Pre-Eclampsia, Preeclampsia Hypertension, Pregnancy-Induced, hypertensive disorder complicating pregnancy, HDCP, gestational hypertension, PE, Hypertension, Pregnancy-Induced, etc. Supplementary Figure S1 shows a sample detailed search strategy for PubMed. Any discrepancies in the literature search were referred to a third party (RH) for resolution.

### 2.2. Literature screening and data extraction

The titles of the articles were read first. After excluding irrelevant literature, the abstract and full text were read to determine inclusion. If necessary, the authors of the original studies were contacted via email and telephone to obtain information not determined but essential for our review.

The inclusion criteria included (1) the diagnostic test for PE; (2) the index test of MPV; (3) guidelines (30, 31, 32), obstetrics and gynecology (33), and clinical diagnostic criteria as the reference standard; and (4) calculation of true positives (TP), false positives (FP), false negatives (FN), and true negatives (TN).

The exclusion criteria included (1) reviews, case reports, letters, conference abstracts, and editorials; (2) non-human studies; (3) unavailable critical information; (4) case or control group sample size less than 10; and (5) duplicate publications.

The data extracted included information on the first author, year of publication, study region, reference standard, study type, gestational week at sampling, sample size, sample source, MPV measurement method, MPV level, MPV cut-off, and TP, TN, FP, FN data. Two investigators (DY and SL) independently screened, extracted, and cross-checked the literature. Disagreements were resolved through discussion or consultation with a third party.

### 2.3. Quality assessment

The risk of bias was evaluated using RevMan 5.3 software. Two investigators (DY and YD) assessed the risk of bias by employing the Quality Assessment of Diagnostic Accuracy Studies 2 (QUADAS-2) tool and cross-checked the results. Any disagreement was submitted to a third party for negotiation.

### 2.4. Statistical analysis

This meta-analysis was performed utilizing RevMan 5.3, Meta Disc 1.4, and Stata 16.0 software. Heterogeneity among the studies was analyzed using the *χ*^2^ test (test level *α* = 0.1), combined with the *I*^2^ value to determine the magnitude of heterogeneity quantificationally. The *I*^2^ values of 25%, 50%, and 75% were considered low, medium, and high heterogeneities, respectively. If there was no statistical heterogeneity among the findings, a fixed effect model was used. If statistical heterogeneity was observed, the source of heterogeneity was analyzed, the effect of obvious clinical heterogeneities was excluded, and a random effect model was further applied to the meta-analysis.

The diagnostic value of MPV was calculated, including the pooled sensitivity (SEN), specificity (SPE), negative likelihood ratio (−LR), positive likelihood ratio (+LR), diagnostic odds ratio (DOR), and area under the summary receiver operating characteristic curve (SROC-AUC). Heterogeneity was categorized into threshold and non-threshold effects, and non-threshold effects were investigated using subgroup analysis. Spearman correlation analysis was employed to check for threshold effects, suggesting no threshold effect if *P* > 0.05 and heterogeneity because of threshold effect if *P* < 0.05. SEN analyses were conducted to estimate the effect of each study on the pooled measures. Publication bias was assessed by the funnel plot. *P* < 0.05 was considered statistically significant.

## 3. Results

### 3.1. Study selection

The works of literature were selected according to the PRISMA diagram. All literature that met the criteria up to 13 March 2023 was included, with a total of 1,036. After removing duplicates, a sum of 528 studies remained to be screened.

**Figure 1:**
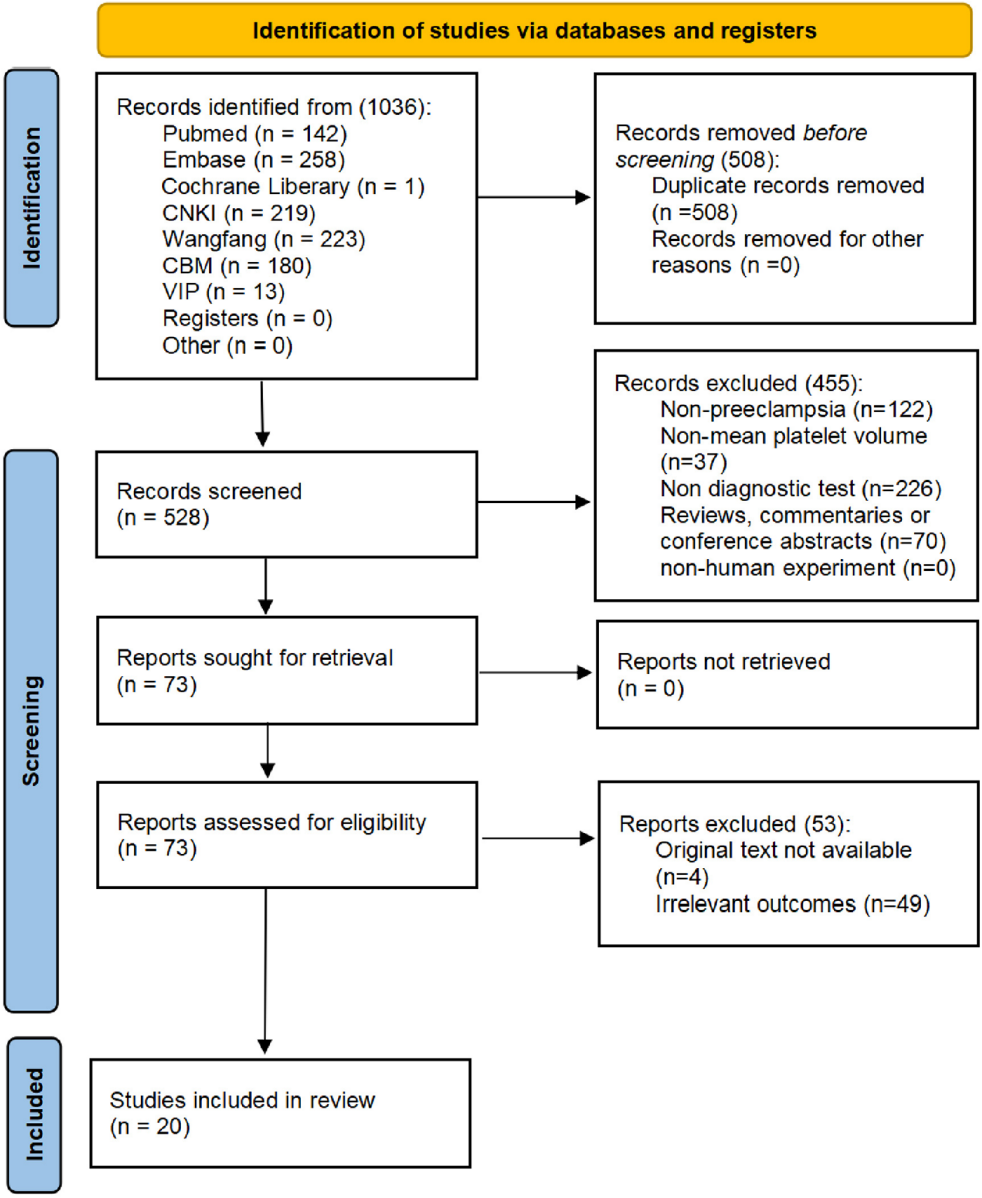
Selection flow chart.

After reading the title or abstract, 455 articles were excluded for not satisfying the inclusion criteria. A total of 49 articles were excluded because the data of the four-compartment table for diagnosing PE by MPV were inaccessible after reading the full text. The full texts of four articles were unavailable. Finally, a total of 20 literature studies (27, 34–52), including 22 studies, were employed in the analysis. Figure 1 shows the screening flow chart.

### 3.2. Study characteristics

This systematic review included 20 publications containing 22 studies. A total of 2,637 cases of PE and 17,500 cases of control were included. Among the 20 literature studies included, seven were performed in China (34, 37–39, 42, 44, 49), five in Turkey (36, 46, 47, 50, 52), two in Ethiopia (35, 41), two in Egypt (43, 48), and four in India (40), Korea (27), Belgium (45), and Iran (51), respectively. Table 1 shows the basic characteristics of the included studies.

**Table 1:** The basic characteristics of the thirteen included studies in the systematic review.

Studies (year)	Country	Study design	Reference standard	No. of P (M and S)/C	Age (years)		Gestational week at sampling		MPV value (fl)		TP	FP	TN	FN	Cut-off
					Pre-eclampsia group	Control group	Pre-eclampsia group	Control group	Pre-eclampsia group	Control group					
Liu 2022 (34)	China	Retro	Guideline^a^	60/60	29.58 ± 5.06^b^	28.28 ± 5.20	12.46 ± 1.19	12.57 ± 0.68	9.54 ± 1.13	8.87 ± 0.95	54	37	23	6	8.545
Walle 2022 (35)	Ethiopia	NR	ACOG	63/63	28.1 ± 4.61	27.5 ± 4.77	34.1 ± 4.4	33.8 ± 4.8	13.47 ± 1.43	11.27 ± 0.92	53	8	55	10	≥12.10
Oğlak 2021 (36)	Turkey	Retro	ACOG	201 (94 and 107)/100	M: 28.3 ± 7.4; S: 28.7 ± 6.8	27.4 ± 6.1	M: 7.2 ± 1.1; S: 7.4 ± 1.3	7.4 ± 1.2	M: 10.6 ± 2.3; S: 11.2 ± 1.4	10.3 ± 1.3	128	35	65	73	10.65
Li 2021 (37)	China	NR	Unclear^c^	92 (54 and 38)/178	32.0 (28.0–31.0)^d^	31.0 (27.0–34.0)	20–24 weeks		M: 10.7 (9.6–11.3); S: 11.8 (9.9–13.5)	9.2 (8.0–10.7)	77	25	153	15	10.5
Yang 2021 (38)	China	NR	Obstetrics and gynecology	162/113	29.93 ± 5.7	29.23 ± 4.68	34.1 ± 4.4	33.8 ± 4.8	10.50 (9.48–11.40)^d^	9.90 (9.40–10.75)	68	24	89	94	10.85
Wang 2021 (39)	China	Retro	Obstetrics and gynecology	100 (40 and 60)/50	M: 28.38 ± 1.2; S: 28.41 ± 1.23	28.12 ± 1.3	M: 38.61 ± 1.0; S: 38.59 ± 1.03	31.45 ± 1.4	M: 9.75 ± 1.04; S: 10.34 ± 1.1	8.69 ± 0.2	13	5	45	87	9.35
Bhamri 2019 (40)	India	Prosp	ACOG	83 (61 and 22)/333	26.43 ± 3.47	25.71 ± 2.64	11–14 weeks		11.41 ± 1.57	10.71 ± 1.81	57	147	186	26	>10.550
Tesfay 2019 (41)	Ethiopia	NR	Unclear^c^	79 (35 and 44)/140	M: 25.20 ± 3.5; S: 25.64 ± 5.26	25.64 ± 3.9	M: 34.51 ± 4.48; S: 34.61 ± 3.99	35.99 ± 3.58	M: 11.5 ± 2.1; S: 12.3 ± 1.7	8.4 ± 0.9	66	19	121	13	>9.45
Wang 2019 (42)	China	Retro	Obstetrics and gynecology	91 (34 and 57)/54	29.1 ± 3.0	26.7 ± 4.3	Antepartum		10.8 ± 1.3	9.6 ± 0.9	62	7	47	29	10.35
Kim 2018 (27)	Korea	Retro	ACOG	353 (126 and 227)/471	M: 32.8 ± 4.6; S: 32.9 ± 4.3	33.3 ± 4.1	M: 36.1 ± 3.6; S: 33.2 ± 3.7	38.7 ± 1.0	M: 9.5 ± 0.1; S: 9.8 ± 0.1	8.8 ± 0.07	131	92	379	222	9
Rezk 2018 (43)	Egypt	Prosp	Unclear^c^	286/9236	30.9 ± 9.9	31.2 ± 9.8	18–20 weeks		9.89 ± 0.8	8.51 ± 0.85	272	3076	6160	14	9.55
Chen 2017 (44)	China	Retro	ACOG	125/188	29.06 ± 4.36	28.87 ± 3.98	27–41 weeks		10.40 ± 2.63	9.49 ± 1.35	96	66	122	29	>10
Mannaerts 2017 (45)	Belgium	Retro	ACOG	118/1495	28.91 ± 4.91	30.20 ± 5.19	Before 20 weeks		8.06 ± 0.87	8.64 ± 1.17	79	653	842	39	8.15
				59/138	28.03 ± 5.06	31.96 ± 4.50	Antepartum		9.51 ± 1.21	8.90 ± 1.17	41	69	69	18	8.85
Gezer 2016 (46)	Türkiye	Retro	ACOG	137/150	28.3 ± 5.98	27.5 ± 5.01	7–14 weeks		8.38 ± 1.12	9.02 ± 1.08	86	53	97	51	8.6
Emel Kurtoglu 2016 (47)	Türkiye	Retro	ACOG	150 (29 and 121)/100	28.9 ± 7.0	30.4 ± 5.3	25–41 weeks		9.0 (5.9–14.2)^e^	8.3 (5.9–12.1)	102	48	52	48	8.35
Nooh 2015 (48)	Egypt	NR	ACOG	192/2621	NR	NR	24–28 weeks		NR	NR	178	341	2280	14	>9.5
Han 2014 (49)	China	Retro	ACOG	94 (53 and 41)/79	M: 27.2 ± 1.9; S: 27.5 ± 1.6	26.7 ± 2.3	M: 80.5 ± 3.2; S: 78.4 ± 2.8^f^	81.7 ± 3.5	NR	NR	87	62	17	7	8.95
Kanat-Pektas 2014 (50)	Türkiye	Prosp	ACOG	15/164	25.2 ± 4.1	26.2 ± 5.3	12.6 ± 0.7	12.6 ± 0.8	11.0 ± 1.2	10.2 ± 0.9	10	59	105	5	10.5
Kashanian 2013 (51)	Iran	Prosp	Unclear^c^	35/269	27.7 ± 6.1	26.9 ± 5.4	10.5 ± 1	10.6 ± 1.2	10.2 ± 1.06	9.68 ± 1.09	21	54	215	14	11.1
							26–28 weeks		10.16 ± 1.23	9.62 ± 1.12	20	49	220	15	10.5
Dundar 2008 (52)	Türkiye	NR	ISSHP	107/1229	28.3 ± 4.3	27.2 ± 6.1	24–28 weeks		NR	NR	83	172	1057	24	>8.5

NR, no report; No. of P (M and S)/C, number of pre-eclampsia patients (mild pre-eclampsia/severe pre-eclampsia)/control; Prosp, prospective; Retro, retrospective; M, mild pre-eclampsia; S, severe pre-eclampsia; FN, false negative; FP, false positive; TN, true negative; TP, true positive.

^a^ Guidelines for Diagnosis and Treatment of Hypertensive Diseases during Pregnancy (2020).

^b^ Mean ± standard deviation.

^c^ Unclear: clinical diagnostic criteria were used, but the names of the criteria were not specified.

^d^ Medians (quartiles).

^e^ Median (min–max).

^f^ Early pregnancy test day.

### 3.3. Quality assessment

**Figure 2:**
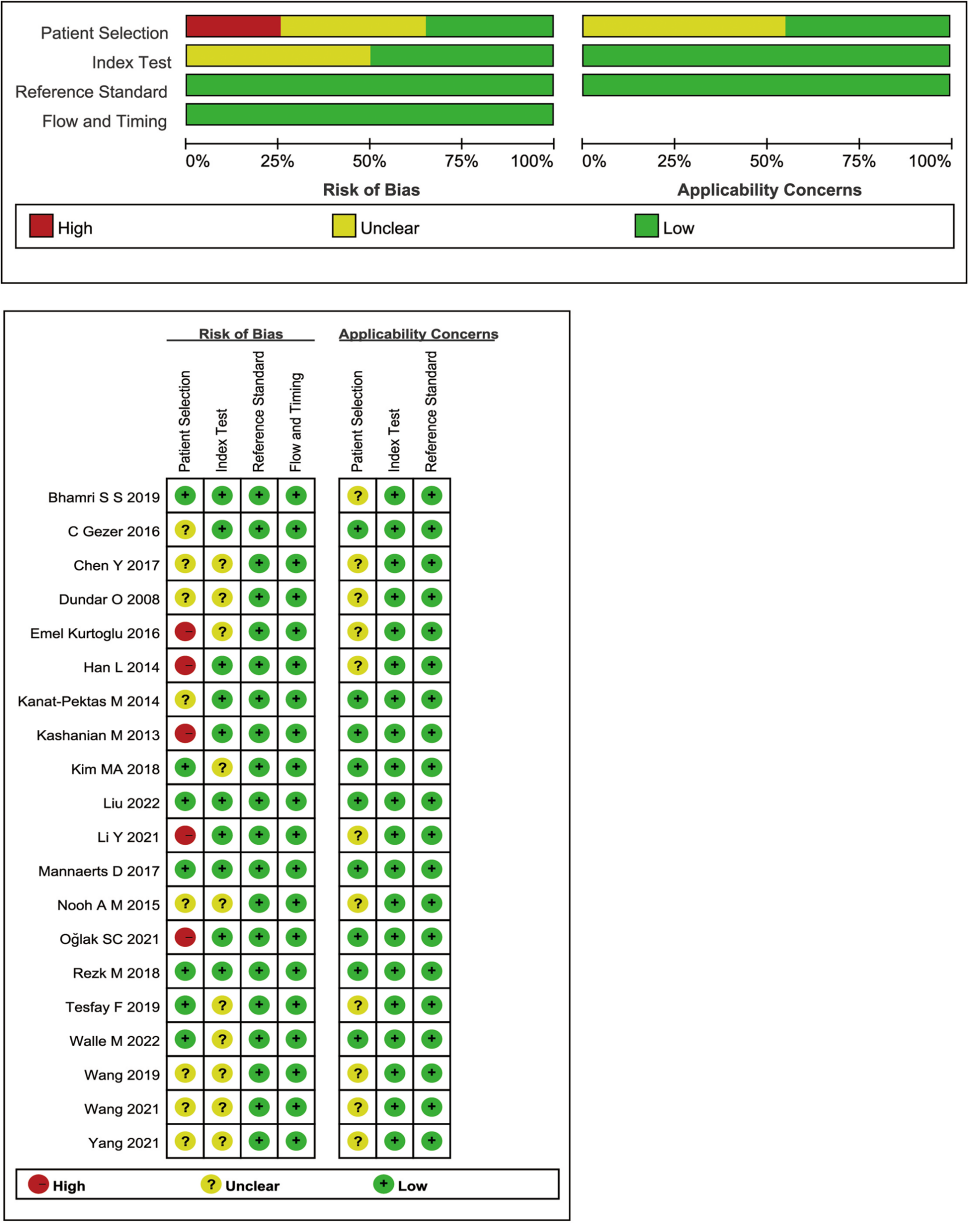
Quality assessment result of the meta-analysis (QUADAS-2).

Figure 2 summarizes the QUADAS-2 quality evaluation of the 20 publications included. 14 studies had (unclear) risk of bias in patient selection, which was due to the lack of (not explicitly reported) whether all females tested by MPV were consecutively or randomly entered, the study applied a case-control design, or was not clearly reported. The bias in the index test and reference standard were attributed to the unclear timing of the MPV threshold setting and the lack of reporting on the use of blinding. The results indicated that the quality of the identified studies was generally satisfactory (Figure 2). In addition, SEN analyses were performed to estimate the impact of each study on the overall, omitting one study at a time, and no outliers were found (Supplementary Figure S2).

### 3.4. Meta-analysis

The Spearman correlation analysis of 22 studies within 20 literature sources used the SEN logarithm with the (1-SPE) logarithm, with a value of 0.178, *P *= 0.428. The *I*^2^ statistics value for SEN and SPE of the pooled studies were 96.6% and 98.2%, respectively, indicating heterogeneity from non-threshold effects.

**Figure 3:**
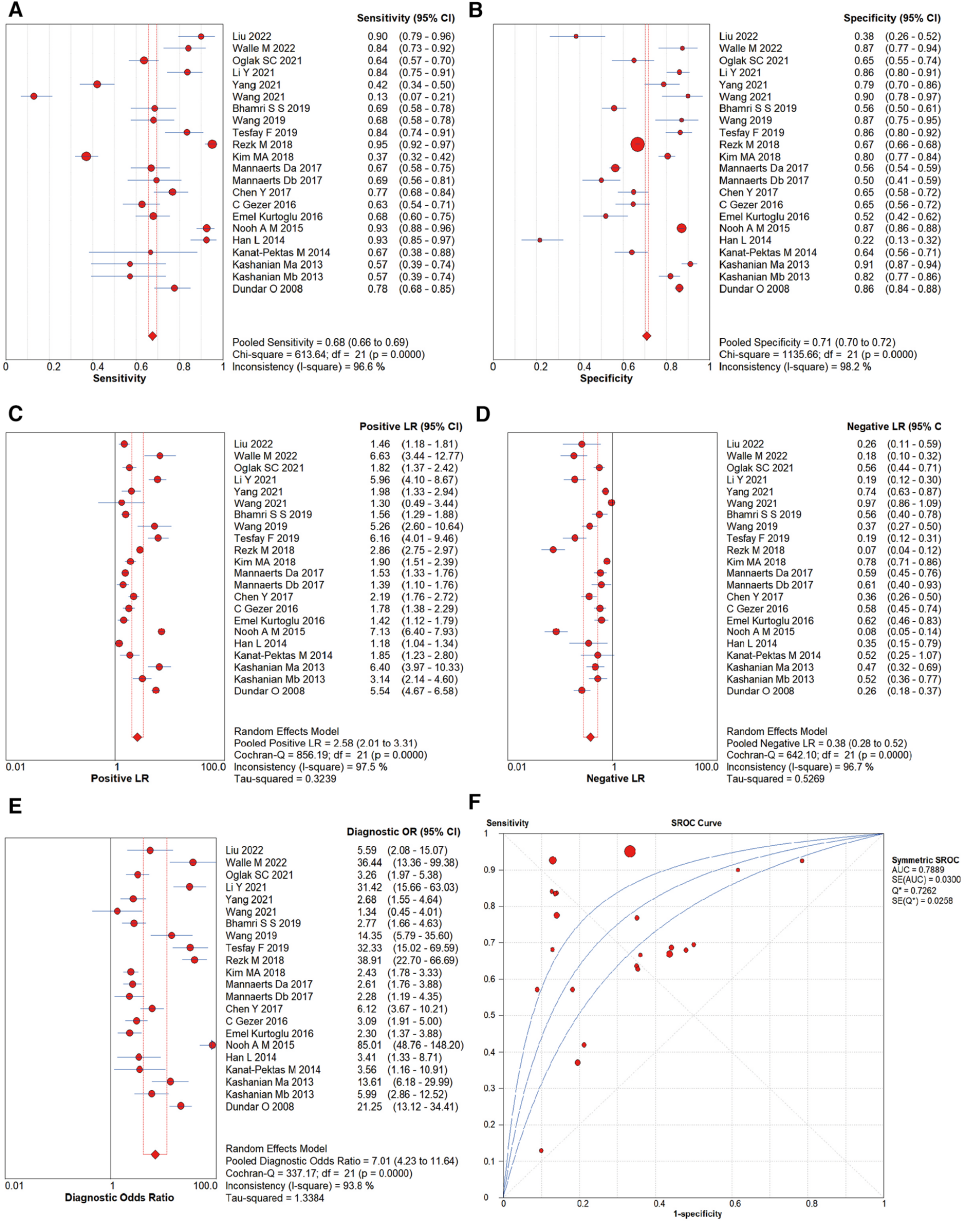
Forest plots of diagnostic performance of MPV for PE: (**A**) sensitivity, (**B**) specificity, (**C**) negative likelihood ratio, (**D**) positive likelihood ratio, (**E**) DOR, and (**F**) area under the SROC curve. The first author's name and year of each study are listed.

By performing a meta-analysis using the random effect model, the overall diagnostic accuracy of MPV for PE was as follows: SEN was 0.707 [95% confidence interval (CI) (0.670–0.743)], SPE was 0.639 [95% CI (0.611–0.667)], +LR was 1.844 [95% CI (1.389–2.447)], −LR was 0.533 [95% CI (0.466–0.610)], and DOR was 4.026 [95% CI (2.727–5.943)] (Figures 3A–E). Figure 3F shows the SROC curve for the diagnostic accuracy of MPV for PE. Based on the SROC, AUC, and Q* indices, the diagnostic accuracy was calculated, and the outcomes showed that the AUC and Q* indices were 0.7889 [standard error (SE) of 0.0300] and 0.7262 (SE of 0.0258).

Studies that sampled MPV measurements before 16 weeks of gestation were selected from 22 studies for the meta-analysis. Seven studies (34, 36, 40, 46, 49, 50, 51) were entered, showing a pooled SEN of 0.707 [95% CI (0.670–0.743)], SPE of 0.639 [95% CI (0.611–0.667)], +LR of 1.844 [95% CI (1.389–2.447)], −LR of 0.533 [95% CI (0.466–0.610)], DOR of 4.026 [95% CI (2.727–5.943)], SROC-AUC of 0.7278 (SE of 0.0294), and Q* indices of 0.6753 (SE of 0.0239).

The cut-off values for MPV in the included literature studies were categorized for analysis. Seven studies (34, 45–47, 49, 52) used the 8–9 fl range as the cut-off, five studies (39, 41, 27, 43, 48) used the 9–10 fl range, eight studies (36–38, 40, 42, 44, 50, 51) used the 10–11 fl range, and two studies used the 11–12 fl (51) and >12 fl (35) ranges as the cut-off, respectively. Since only two reports had a cut-off value of 11–12 and >12 fl, the SROC analysis was performed for studies with a cut-off range of 8–9, 9–10, or 10–11 fl.

In the cut-off range of 8–9 fl for MPV, the pooled SEN, SPE, AUC, and Q* indices for the diagnosis of PE were 0.734 [95% CI (0.700–0.766)], 0.663 [95% CI (0.647–0.680)], 0.7363, and 0.6822, respectively. The pooled SEN, SPE, AUC, and Q* indices for the 9–10 fl range were 0.653 [95% CI (0.623–0.683)], 0.718 [95% CI (0.710–0.726)], 0.8856, and 08162. In 10–11 fl, the pooled SEN was 0.644 [95% CI (0.610–0.677)], SPE was 0.706 [95% CI (0.681–0.729)], AUC was 0.7623, and Q* index was 0.7037.

### 3.5. Subgroup analysis

To examine the heterogeneity of the 22 studies identified, six subgroups were distinguished. The diagnostic accuracy was similar between study areas and sample sizes, with significant differences between gestational week at sampling, cut-off, reference standard, and study design, as detailed in Table 2. (i) In the group of gestational weeks at sampling, the diagnostic accuracy of MPV for PE after 20 weeks of gestation [DOR = 8.661, 95% CI (4.156–18.048)] was higher than the predictive value before 20 weeks of gestation [DOR = 5.165, 95% CI (2.582–10.332)] (Supplementary Figures S3, S4). (ii) Compared with the seven studies employing MPV <9 fl as the cut-off [DOR = 4.021, 95% CI (2.034–7.951), AUC (SE) = 0.7363 (0.0543)], the 15 studies with MPV cut-off ≥9 fl had a DOR = 9.129, 95% CI (4.614–18.059) along with an AUC (SE) = 0.8421 (0.0294), showing a better overall accuracy, suggesting that the capacity to distinguish between pregnant women with PE is stronger when the cut-off is ≥9 fl than when the MPV < 9 fl. (iii) When a different reference standard was used to diagnose PE in pregnant women, the pooled diagnostic precision was lower in 12 studies using ACOG [DOR = 4.843, 95% CI (2.645–8.867)] than in five studies using the clinical criteria [DOR = 20.367, 95% CI (9.970–41.608)]. (iv) The diagnostic value of prospective studies had a higher AUC (SE) = 0.8053 (0.0589) than that of retrospective studies AUC (SE) = 0.6889 (0.0228) at the time of differentiating between study designs.

**Table 2:** Summary estimates of sensitivity, specificity, +LR, −LR, DOR, and SROC curve (AUC) of MPV for the identification of PE in different subgroups.

Variables		No.	Sensitivity (95% CI)	Specificity (95% CI)	+LR (95% CI)	−LR (95% CI)	DOR (95% CI)	SROC curve AUC ± SE
Total		22	0.676 (0.658–0.694)	0.710 (0.703–0.717)	2.576 (2.006–3.308)	0.381 (0.277–0.523)	7.012 (4.226–11.636)	0.7889 ± 0.0300
Gestational week at sampling	Before 20 weeks	9	0.771 (0.744–0.796)	0.651 (0.643–0.660)	1.939 (1.332–2.823)	0.393 (0.251–0.613)	5.165 (2.582–10.332)	0.7467 ± 0.0490
	After 20 weeks	13	0.616 (0.591–0.640)	0.835 (0.825–0.845)	3.187 (2.075–4.897)	0.374 (0.247–0.568)	8.661 (4.156–18.048)	0.8387 ± 0.0378
Sample size	<350	16	0.656 (0.632–0.680)	0.721 (0.701–0.740)	2.469 (1.853–3.290)	0.432 (0.321–0.581)	5.996 (3.742–9.608)	0.7700 ± 0.0326
	≥350	6	0.702 (0.675–0.729)	0.709 (0.701–0.716)	2.836 (1.788–4.498)	0.277 (0.100–0.766)	10.268 (3.007–35.064)	0.8169 ± 0.0665
Cut-off	<9	7	0.734 (0.700–0.766)	0.663 (0.647–0.680)	1.756 (1.151–2.678)	0.470 (0.353–0.624)	4.021 (2.034–7.951)	0.7363 ± 0.0543
	≥9	15	0.654 (0.632–0.676)	0.721 (0.713–0.728)	3.137 (2.354–4.181)	0.352 (0.227–0.547)	9.129 (4.614–18.059)	0.8421 ± 0.0294
Country	China	7	0.631 (0.595–0.666)	0.687 (0.652–0.721)	2.246 (1.367–3.690)	0.412 (0.232–0.731)	5.819 (2.729–12.405)	0.7657 ± 0.0525
	Other countries	15	0.693 (0.672–0.714)	0.711 (0.704–0.718)	2.735 (2.035–3.677)	0.366 (0.244–0.551)	7.658 (3.994–14.682)	0.7995 ± 0.0380
Reference standard	ACOG	12	0.659 (0.635–0.682)	0.723 (0.712–0.735)	2.052 (1.329–3.168)	0.434 (0.308–0.611)	4.843 (2.645–8.867)	0.7436 ± 0.0487
	Obstetrics and gynecology	3	0.405 (0.353–0.458)	0.834 (0.778–0.881)	2.446 (1.163–5.141)	0.652 (0.401–1.062)	3.768 (1.096–12.953)	0.8642 ± 0.1015
	Guideline	1	0.900(–)	0.390(–)	—	—	—	0.647
	ISSHP	1	0.78(–)	0.86(–)	—	—	—	0.748
	Clinical diagnostic criteria	5	0.863 (0.831–0.892)	0.684 (0.674–0.693)	4.572 (2.401–8.705)	0.233 (0.103–0.528)	20.367 (9.970–41.608)	0.8927 ± 0.0198
Study design	Prospective	5	0.835 (0.797–0.868)	0.673 (0.664–0.682)	2.703 (1.806–4.047)	0.351 (0.146–0.848)	8.062 (2.477–26.245)	0.8053 ± 0.0589
	Retrospective	11	0.591 (0.565–0.616)	0.609 (0.591–0.627)	1.659 (1.405–1.959)	0.550 (0.437–0.693)	3.279 (2.468–4.356)	0.6889 ± 0.0228
	NR	6	0.755 (0.722–0.787)	0.864 (0.854–0.874)	5.189 (3.710–7.260)	0.219(0.081–0.593)	23.481(8.211–67.146)	0.9293 ± 0.0073

### 3.6. Publication bias

**Figure 4:**
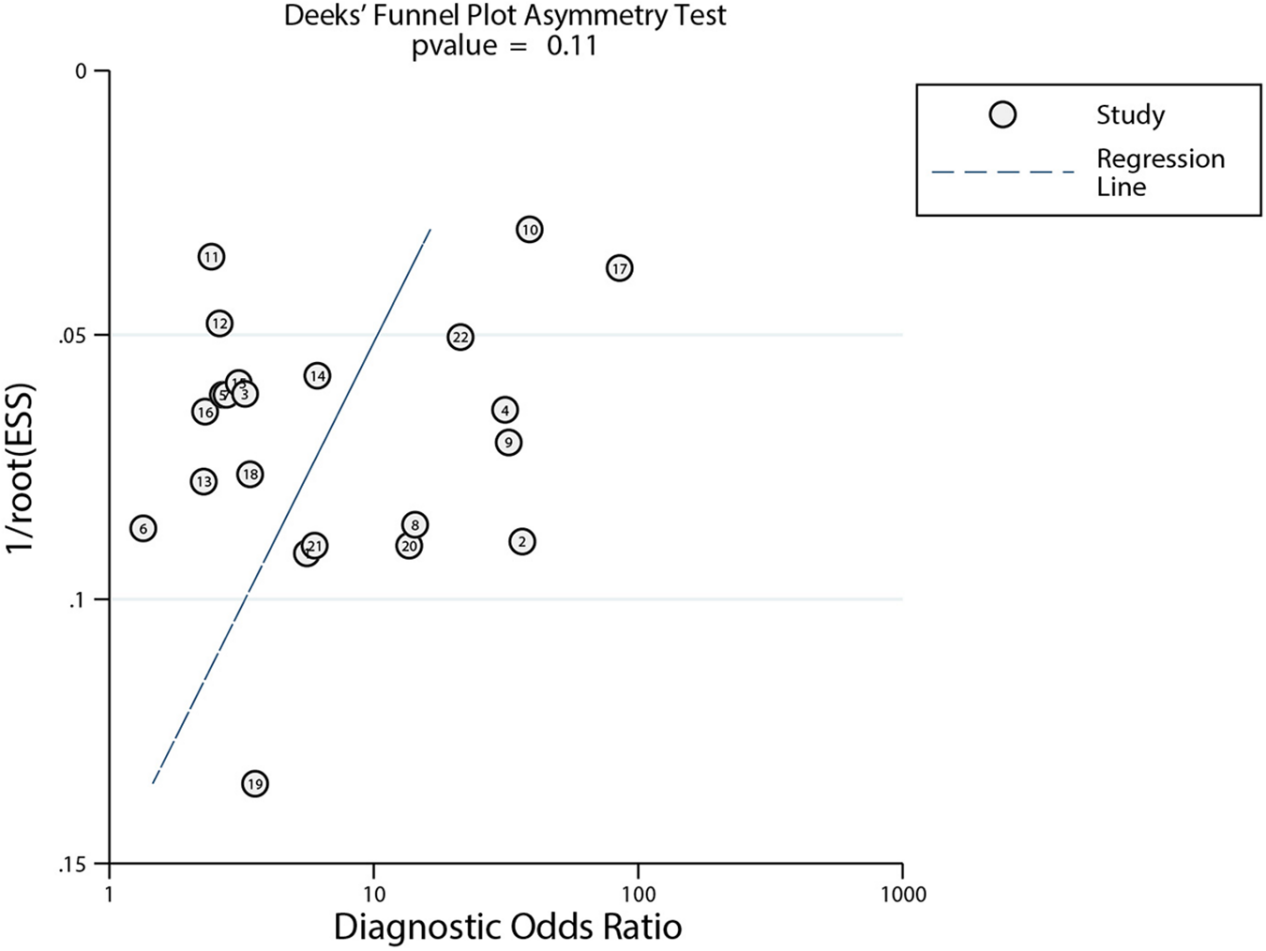
Deek's funnel plots.

After plotting Deek's funnel plot to test for publication bias, the results showed that the left–right distribution of study points was generally symmetrical, with *P *= 0.11, indicating that the likelihood of publication bias in our review was low (Figure 4).

## 4. Discussion

PE remains one of the primary contributors to maternal mortality globally, particularly in underdeveloped regions and countries. The only option to address the disease is to terminate the pregnancy. A substantial amount of evidence shows that taking aspirin before 16 weeks of pregnancy in women with high-risk factors can prevent PE (23). An early predictive diagnosis of PE has not yet become widespread, although guidelines suggest that clinical risk factors, blood pressure, uterine artery pulsatility index, and PlGF can be used as risk markers for screening for PE (2). A meta-analysis suggests that the sFlt-1/PlGF ratio can be used to predict the development of future PE or exclude PE in high-risk pregnancies (53). In fact, there are no reliable predictors or co-predictors of the development of PE in early or mid-pregnancy. In addition, testing methods can be financially prohibitive in less developed areas, making it difficult to detect and focus on the disease until severe clinical symptoms and multi-organ dysfunction are present, hindering the clinicians from preventing the disease early and providing timely medical intervention. Using convenient and cost-effective methods for predicting and diagnosing PE early remains a clinical challenge. To forecast PE and enable serial prevention and earlier treatment, more attention should be paid to readily available risk markers. Clinical reports of the diagnostic accuracy of MPV for PE cut-off vary, and no systematic reviews evaluated the accuracy of MPV in predicting and diagnosing PE, which is why we undertook this study.

One possible pathogenesis of PE is the placental release of pro-inflammatory mediators through the activation of immune and coagulation mechanisms, leading to endothelial dysfunction and increased platelet activation (4, 54). MPV is an indicator of platelet function activation and a routine parameter of CBC, which is easy and inexpensive to measure. It has been suggested that MPV rises progressively during pregnancy, with higher MPV values in PE than in normotension. MPV will increase as the severity of the disease increases (52, 55, 56). However, the diagnostic utility of MPV has been inconclusive. Our study is the first systematic review to explore the diagnostic value of MPV in PE. Based on a large number of original studies, including a sample of 2,637 patients with PE, the results of the meta-analysis revealed an acceptable SEN and moderate SPE, while the AUC and Q* indices were 0.7889 and 0.7262, which indicated that MPV has a certain diagnostic value for PE.

Based on recommendations on using aspirin to prevent PE, predicting PE before 16 weeks of gestation or even earlier and screening pregnancies at high risk of PE should be focused. A large sample of studies hinted that MPV has the potential to predict PE before the onset of the disease (57). Our review analyzed the predictive value of MPV for PE before 16 weeks of gestation, showing that MPV has moderate predictive power for PE in early pregnancy.

As different cut-off ranges were used to diagnose PE using MPV in our selected studies, this study analyzed the cut-off in various sections, proposing to ascertain the cut-off with the greatest diagnostic value. Results showed that SEN, SPE, AUC, and Q* indices for PE diagnosis were higher when the cut-off of MPV was in the range of 9–10 fl compared with other intervals. Three of the five studies in that range were around 9.5 fl. We infer that an optimal cut-off for the diagnostic accuracy of MPV for PE is 9.5 fl.

For the quality of the study, a large portion of the included literature studies used a case–control design, so selection bias cannot be eliminated. In addition, as the use of blinding is not adequately reported, the potential bias in the index test should be evaluated.

Heterogeneity is a potential problem for almost every meta-analysis, as its presence may partially reduce the stability of the study. The Spearman correlation analysis implied that heterogeneity in our review was attributable to non-threshold effects, so we conducted subgroup analyses of all 22 studies to explore the sources of heterogeneity. According to the original inclusion and exclusion criteria, no particular population was excluded because of limitations in the number of studies. Based on the diagnostic criteria for PE, we divided the groups using a threshold of 20 weeks of gestation. The subgroup analysis showed a superior diagnostic value of MPV for PE when sampled at >20 weeks of gestation, MPV ≥ 9 fl, using the clinical criteria as the reference standard and prospective design. Since the original study did not report information on ethnicity, race, education, economic status, treatment or not before enrollment, and other factors that might influence the diagnostic value of MPV for PE, these variables were not analyzed. In future studies, considering the impact of these risk factors on the diagnostic value of MPV may define the most appropriate diagnostic range for MPV. There was no publication bias among the enrolled studies.

Based on clinical features, PE can be classified into non-severe and severe. A prospective cohort study testing MPV at 24 weeks of gestation revealed an excellent predictive accuracy for mild PE, with outcomes suggesting a SEN of 0.78, SPE of 1.0, and AUC of 0.936 when MPV > 9.7 fl (58). As for the diagnostic value of MPV for severe PE, the study by Freitas et al. (59) in late pregnancy indicated a SEN and SPE of 0.5172 and 0.8276, respectively, with an AUC of 0.72, which did not accurately screen for severe PE. When the MPV was used to differentiate the severity of PE, it had a SEN of 0.875 and a SPE of 0.853, implying that it could distinguish disease severity better (60). However, the ability of MPV to differentiate the severity of PE could not be analyzed using the systematic review approach because of the limited number of studies. Furthermore, the limited number of studies dividing PE into early-onset and late-onset makes it impossible to obtain data on the diagnostic value of MPV in these two clinical subtypes.

Currently, CBC has been emphasized in studies for the forecasting and detection of PE because of its simplicity; however, its availability still requires exploration. A systematic review by Walle et al. (16) showed that platelet count (PC) was dramatically decreased in PE. A recent comparative cross-sectional study (61) showed that PC has a strong diagnostic value for pregnancy-induced hypertension (PIH), with a SEN of 96.7%, SPE of 90%, and AUC of 0.995. Meanwhile, its AUC for distinguishing between severe and mild PIH was 0.947, which implies that PC is valuable for predicting the development of PIH and determining its severity. As a helpful marker of systemic inflammatory response, red cell distribution width (RDW) had markedly higher levels in PE and was notably higher in SPE than MPE (62). A prospective case–control study by Sachan et al. (63) revealed that the RDW had a SEN of 85.3%, SPE of 49.0%, and AUC of 0.751 in differentiating healthy normotensive pregnant women from non-severe PE. The neutrophil-to-lymphocyte ratio (NLR) is an inflammation-related indicator that can be calculated from the CBC. A meta-analysis reported that NLR is elevated in PE, especially in SPE, with an AUC of 0.82 (64, 65). Similar to NLR, the platelet-to-lymphocyte ratio (PLR) is also potentially predictive of PE, with a meta-analysis showing that PLR declines with PE severity and has an AUC of 0.7296 for the diagnosis (66). The above laboratory indices, together with MPV, are derived from CBC, and all indices have acceptable individual diagnostic values for PE, However, clinical studies on the predictive ability of PE are scarce. More high-quality studies are needed for validation.

The limitations of our study include the following: (i) Most of the included studies were diagnosed with confirmed cases and hospital populations, which may produce selective bias for specific regions, ethnicities, languages, or populations. (ii) There was a large heterogeneity between studies; however, our study only analyzed six aspects in subgroups, while differences in ethnicity, race, severity of disease, treatment or not before enrollment, type of instrumentation, and source of reagents may also have contributed to the heterogeneity, but an in-depth analysis was not conducted because of the limitations of the number of studies. (iii) The categories of PE were not differentiated for assessing the diagnostic value.

Our findings support that MPV is a promising biomarker with a good predictive diagnostic value for PE, especially after 20 weeks of gestation. Thus, in the absence of specific laboratory tests, MPV may be used as a predictive diagnostic tool for PE and as an adjunct to the management of PE by administering aspirin to prevent the onset of PE, pumped magnesium sulfate to prevent convulsions, anti-hypertensive medication to decrease blood pressure, and timely intervention for premature delivery. However, to fully elucidate its application in clinical practice and accurately evaluate the role of MPV in early pregnancy, larger prospective, multicenter, cohort studies are needed. Moreover, the onset and severity of PE should be distinguished, and the diagnostic accuracy in the different types of PE should be evaluated. Finally, MPV should be jointly estimated with other markers of PE, particularly those that can be extracted in CBC such as PC and NLR, and a combined predictive diagnostic model should be established to provide the most efficient predictive diagnosis of the disease.

## 5. Conclusion

The results of this meta-analysis suggest that MPV has an acceptable value as a promising, convenient, and affordable marker for the prediction and diagnosis of PE. For MPV, the optimal sampling gestational weeks and a more precise cut-off for its diagnosis of PE should be determined and investigated in depth. In addition, combining MPV parameters with other platelet parameters and measurable factors in CBC may be more important than using a single indicator alone to diagnose PE. Therefore, it is essential to explore the SEN and SPE of MPV in combination with other indicators. In the future, multicenter prospective studies are also needed to assess the role of MPV in different subtypes of PE.

## Data Availability

The original contributions presented in the study are included in the article/Supplementary Material, further inquiries can be directed to the corresponding author.
