# Supplementary figures and images for: Clinical value of mean platelet volume in predicting and diagnosing pre-eclampsia: a systematic review and meta-analysis
Source: Front Cardiovasc Med. 2023 Oct 6;10:1251304. doi: 10.3389/fcvm.2023.1251304 (PMC10587588; doi:10.3389/fcvm.2023.1251304)

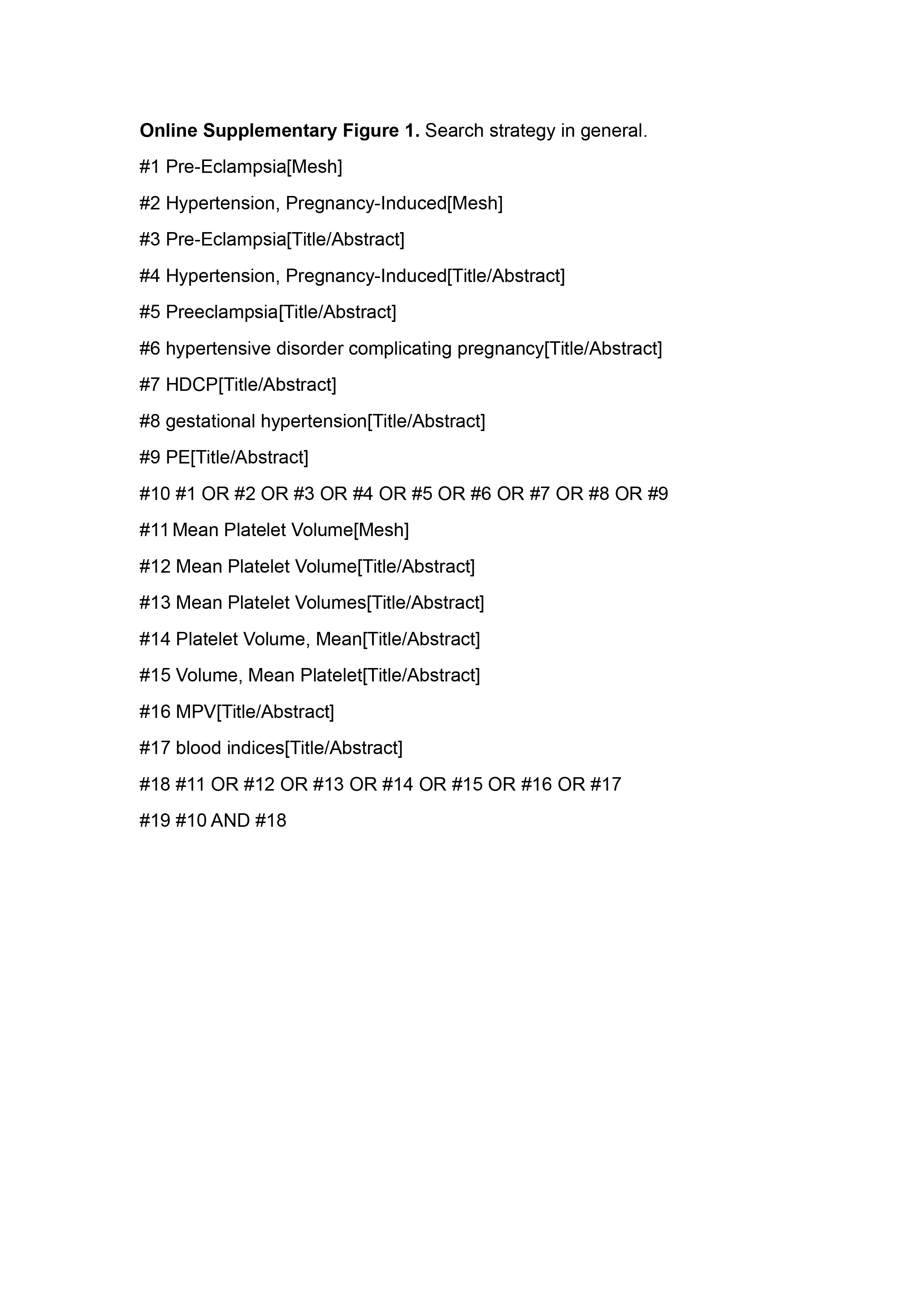

Supplement: Supplementary file 1 [file Image1.jpg]

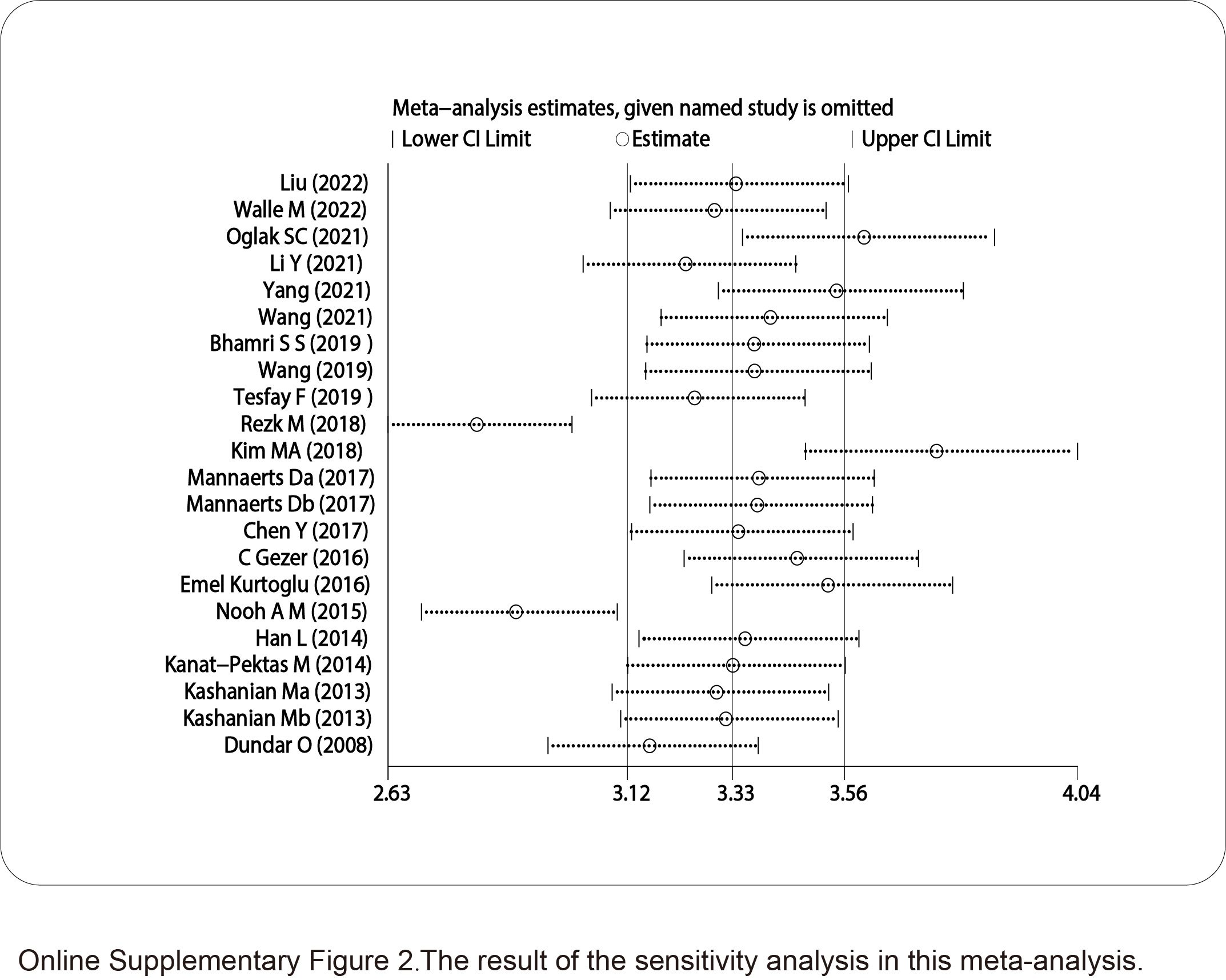

Supplement: Supplementary file 2 [file Image2.jpg]

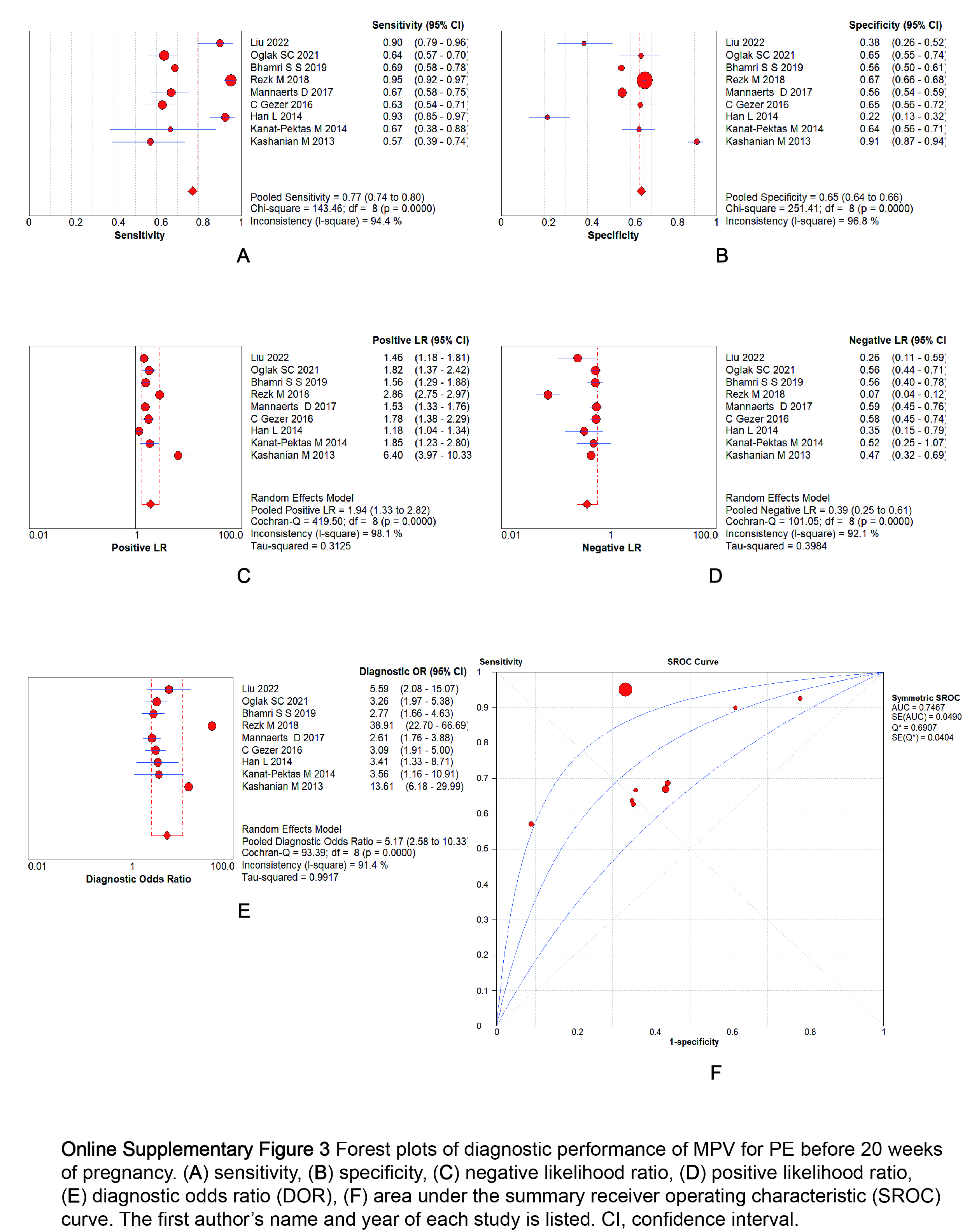

Supplement: Supplementary file 3 [file Image3.jpg]

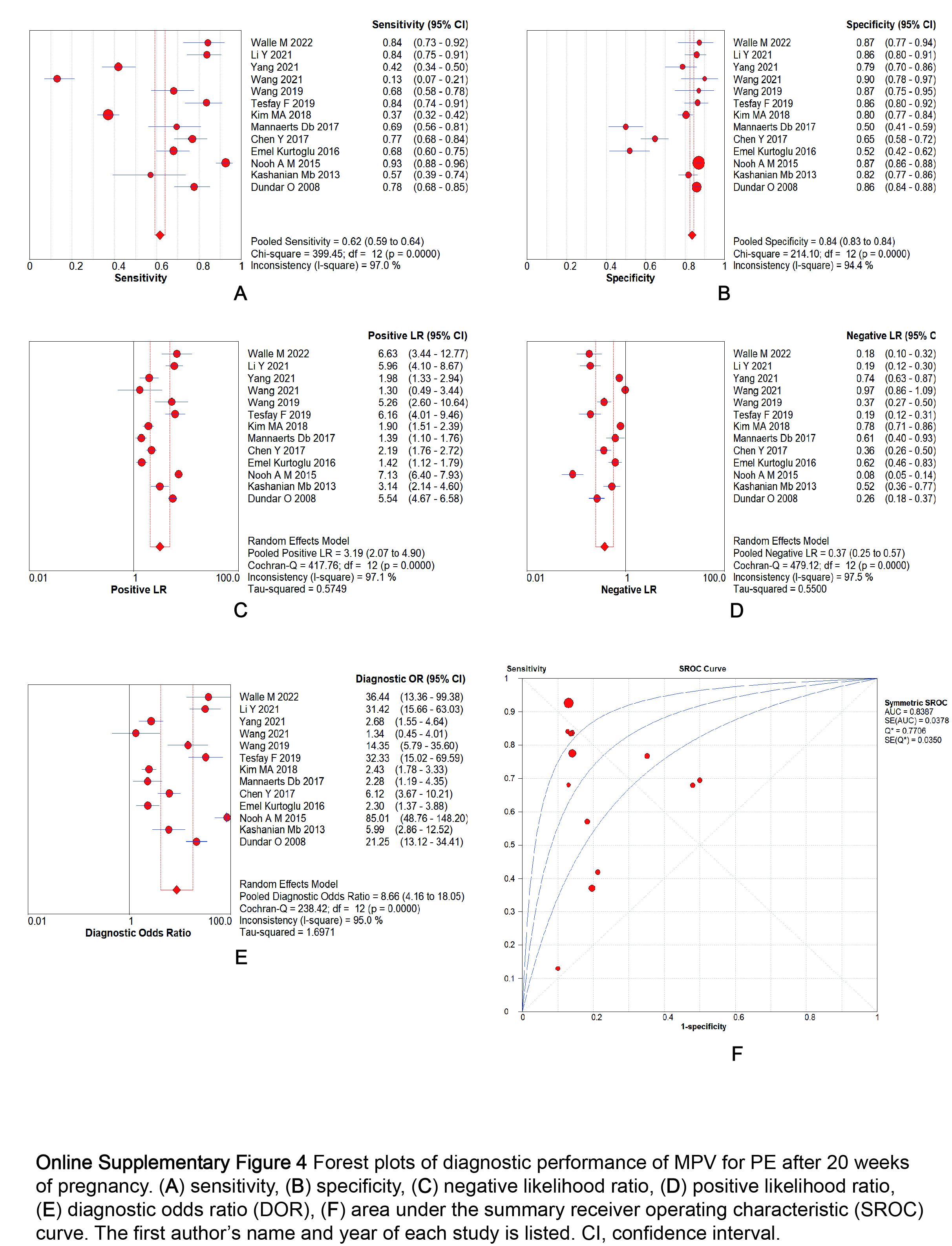

Supplement: Supplementary file 4 [file Image4.jpg]
